# Health literacy and health outcomes in China’s floating population: mediating effects of health service

**DOI:** 10.1186/s12889-021-10662-7

**Published:** 2021-04-08

**Authors:** Hai-YanYu, Wei-Ling Wu, Lin-Wei Yu, Lei Wu

**Affiliations:** 1 7B304, School of Public Health Management, Wenzhou Medical University, Chashan Town, Ouhai District, Wenzhou City, China; 2 Shaoxing College of Arts and Sciences, 508 Huancheng West Road, Shaoxing, China

**Keywords:** Floating population, Health literacy, Health outcomes, Health services

## Abstract

**Background:**

The floating population in China consists primarily of internal immigrants and represents a typical health vulnerable group. Poor health literacy has recently become an obstacle in the accessibility and utilization of health services for the vulnerable population, leading to adverse health outcomes. This study aimed to examine whether health literacy affected health outcomes in China’s floating population and whether health service utilization had a mediating effect between health literacy and health outcomes.

**Method:**

The current study utilized a cross-sectional stratified, multistage, proportional to scale (PPS) study in Zhejiang Province, China, in November and December 2019. In total, 657 valid self-reported questionnaires were recovered and used for data collection. Questionnaires included questions regarding sociodemographic characteristics, health literacy, health outcomes, and health service utilization. Confirmatory factor analysis was used to test questionnaire validity; descriptive statistics were used to understand the demographic characteristics of the floating population; and structural equation modeling was used to determine whether health service utilization mediated health literacy and health outcomes.

**Results:**

We report positive correlations between health literacy, health service utilization, and health outcomes. Mediation analysis demonstrated that health service utilization had partial mediating effects between health literacy and health outcomes. In the relationship between health literacy and health outcomes, the indirect effects of health service utilization accounted for 6.6–8.7% of the total effects.

**Conclusion:**

Complete health literacy, through health care literacy and health promotion literacy, affects the mobile population’s initiative to use health services, which, in turn, affects health outcomes. Thus, improving the health literacy of the floating population will help to improve health outcomes. Furthermore, health service providers should enhance the diversity of health service supply to ensure that the floating population has the external resources to improve personal health literacy.

## Background

Health literacy, or lack thereof, has become a global problem. The World Health Organization has defined health literacy as “the cognitive and social skills which determine the motivation and ability of individuals to gain access to, understand and use information in ways which promote and maintain good health” [1]. Studies have found that health literacy is associated with access to health services, and lower health literacy leads to adverse health behavior and outcomes. Indeed, low health literacy not only directly leads to higher disease prevalence, but also limits individuals’ understanding of health information and health guidance, which further leads to poor disease self-management [2, 3]. Individuals with low health literacy fail to make full use of preventive and medical services, which heightens the cost of eventual hospital stays and medical care [4]. However, there has been little research on health literacy in non-English-speaking countries, and even less on vulnerable populations in those countries, such as China’s “Floating Population”.

The floating population is a concept that was developed under China’s household registration system. Briefly, it refers to people who have left their domicile of origin but find they cannot enjoy the same health services as local populations because of differences in household registration. Notably, internal migration often leads to changes in employment, living environment, and social networks. Internal migration is a typical marker of economic development and social progress, and in the context of rapid ongoing urbanization, large-scale population migration is predicted to be an important factor in China’s continuing socioeconomic development. However, population migration requires healthy citizens, which makes health a prerequisite for the development and survival of the floating population.

Migrant health is affected by various factors, including insufficient health awareness, limited financial capacity, place of residence, and work environment [5].China’s floating population is particularly vulnerable to unhealthy lifestyles due to lack of health literacy. A survey in Zhejiang Province found that the incidence of measles was positively correlated with the proportion of individuals in the floating population, likely due to low vaccination rates among provincial floating populations [6]. Another study found that self-measurement scores of individuals in the floating population were significantly lower than those of the local population in areas of physical health (i.e., fatigue and gastrointestinal symptoms) and psychological health (i.e., anxiety and depression). Additionally, the psychological health of a floating population is typically correlated with how well they can integrate with local residents [7].

Research on migrant health outside of China shows similar findings. A United States-based study found that the risk of cardiovascular disease was higher in the floating population than in the local population [8]. Furthermore, among African immigrant youth in Canada, problems related to regional discrimination, identity, and cultural impact adversely affected psychological health [9]; however, only 13.3% were willing to seek out psychological health services [10]. Immigrants tend to experience heavy mental burdens after settling in a new area [11], and this is certainly true of China’s floating population. As such, it is important to focus on the health problems of this population.

Proposing that the individual is ultimately responsible for his or her health, the “Healthy China” strategy advocates for increasing health literacy, shifting the focus from disease treatment to prevention, and accelerating the adoption of healthy lifestyles. On a global scale, one study found that increased disease-prevention literacy in a rural Turkish population was positively correlated with vaccination rates [12]. Due to low health literacy, China’s floating population is at higher risk for infectious disease transmission. Indeed, a lack of reproductive health knowledge has increased the spread of sexually transmitted diseases [13]. Many youths in the floating population also have irregular diets, excess stress, and a lack of routine medical treatment, all of which could lead to adverse health outcomes. A Korean-based study found a correlation between the health literacy level of migrants and risk factors for type 2 diabetes [14]; poor health literacy is also correlated with HIV infection among the African American population [15]. Another study found that although locals and immigrants face similar mental health problems, immigrants are less likely to use mental health services [16]. Finally, a recent study reported that poor health literacy could act as a barrier to accessing health services among vulnerable populations, thereby resulting in poor health outcomes [17].

Healthcare systems based on place of residence and employment status can make it difficult for migrants to access health services [18], although increased health service utilization (including medical, healthcare, and rehabilitation services) would likely reduce adverse health outcomes in vulnerable populations. One study in particular found that maternal health services significantly reduced mortality rates among pregnant women [19]. However, due to the restrictions of China’s household registration system, the floating population has both low awareness [20] and poor utilization of health services, and subsequent lower vaccination rates compared to local populations [21]. Outside of China, health service utilization by immigrants is similarly affected by health literacy. In New York City, for example, low mental health service utilization rates among elderly Chinese immigrants were shown to increase the risk of mental illness [22]. Additionally, only 45.6% of immigrant subjects in Brazil used some form of medical service [23].

Health literacy and health service utilization both influence health outcomes. However, an effort to improve the initiative of the floating population to utilize health services through an improvement in health literacy has not yet been investigated. We propose researchers and health advocates alike focus on three dimensions of health promotion to improve the health of the floating population: health awareness, health behavior, and supportive environments. Indeed, health awareness and health behavior are both part of the broader health literacy, while a supportive environment refers to external health services. An individual’s health literacy must merge with a supportive external environment to ultimately improve health outcomes. Hence, the current study aimed to examine the relationship between health literacy and health outcomes in China’s floating population and to determine whether health service utilization has a mediating effect in this relationship.

## Method

The data used in this study were obtained from Zhejiang Province, China. Zhejiang province has had the second-largest floating population in China for 19 continuous years (~ 22.57% of China’s 241 million-person floating population). Representative cities Hangzhou, Ningbo, and Wenzhou were selected as study sites due to their robust internal migrant populations. Before conducting the survey, a preliminary test was carried out in which 150 questionnaires were distributed and 148 were collected (recovery rate: 98%). The internal consistency reliability was tested by clonbach consistency coefficient, and the validity was tested by exploratory factor analysis and confirmatory factor analysis. The revised questionnaire was then used to carry out a large-scale survey in November 2019.

The 2017 annual floating population data for Zhejiang Province were used as the sampling frame basis. A stratified, multistage, scale-proportion probability proportional to size (PPS) method was used for sampling. Three sampling sites were randomly selected from Hangzhou, Ningbo, and Wenzhou, and three communities were randomly selected in each sampling site. Researchers selected 20–40 people from the floating population in each selected community according to gender, age, and migration time. The inclusion criteria included people who had been in Zhejiang for at least 1 year before the survey, did not have a registered household address in the region (county, city), and were over age 15 in November 2019. The exclusion criteria included students and transient populations at train stations, harbors, airports, hotels, and hospitals. In order to improve the questionnaire recovery rate, the research team conducted online and offline training for the investigators and respondents and provided on-site guidance and rewards for the investigation. A total of 670 questionnaires were distributed online and offline, and 657 valid questionnaires were recovered (validity rate: 98%).The structural equation model research requires that the ratio of parameters to be estimated in the model should be between 1:5 and 1:10. The structural equation in this paper has 55 parameters to be estimated; thus, the sample size is required to be between 275 and 550. The number of samples recovered was 657, which meets needs of empirical research for the structural equation. Before each subject completed the self-reported questionnaire, the investigator explained the aim of study, the data collection method, and how to complete the survey. The investigators also informed subjects that participation was anonymous and voluntary. This study received ethical clearance from the Ethics Committee of Wenzhou Medical University.

### Measurements

#### Health gain

Health outcomes are used as outcome indicators in the form of self-rated reports. The dependent variables of self-evaluated health, physiological health, and psychological health were used to examine health status in the floating population. The short-form 36 questionnaire (SF-36) from the United States was used to assess *self-evaluated health* [24]; it measures self-perceived health of an individual compared to peers using a five-point Likert scale. A simplified version of the Patient Health Questionnaire (PHQ-15) was used to measure *physiological health*; the Chinese version has shown good validity and reliability [25]. The scale includes questions related to headache, chest pain, and arthralgia, each assessed on a five-point Likert scale. Finally, a simplified version of the Hopkins Symptoms Check List (HSCL) was used for *psychological health*. The scale measures anxiety and depression using a five-point Likert scale; here, the higher the score, the poorer the perceived psychological health of the individual [26].

#### Health services

The health service is effect modifiers. The “2017 Chinese Floating Population Health and Family Planning Dynamic Monitoring Questionnaire” was used to evaluate the level at which health services were accessed by the floating population. The questionnaire includes five content areas: (1) general status of family members, (2) movement trends and residence intention, (3) employment characteristics, (4) basic public health service utilization, and (5) social integration. This study used the basic public health service utilization section, which includes 14 basic public health service items (i.e., health record management, health education, prophylactic vaccination, health management of 0–6-year-old children, health management of pregnant women, health management of elderly people, health management of chronic disease patients (hypertension, diabetes), management of severe mental illness patients, health management of tuberculosis patients, infectious diseases and public health emergency reporting and management services, traditional Chinese medicine health management, health and family planning monitoring services, free contraceptive tools, and health literacy promotion). The Chinese Floating Population Health and Family Planning Dynamic Monitoring Questionnaire is divided into four dimensions of vaccination services, health examination, health education, and physical examination; five-point Likert scales were used to assess health services provided in these four dimensions.

#### Health literacy

The Health literracy is predictors. The European Health Literacy Survey Questionnaire was used to investigate health literacy in the floating population. This questionnaire is multidimensional and has been used to measure health literacy in European populations [27]. The questionnaire is available in three versions that measure health literacy in different ways. Here, we used the HLS-EU-Q47 questionnaire, as it is the most widely used, and has high validity and reliability in Asian contexts [28]. The HLS-EU-Q47 is based on a 4 (information processing: finding, understanding, judging, applying) × 3 (health domain: health care, disease prevention, health promotion) matrix and contains 47 questions that measure health literacy using a four-point Likert scale (1: extremely difficult to 4: extremely easy).

### Data analysis

**Fig. 1:**
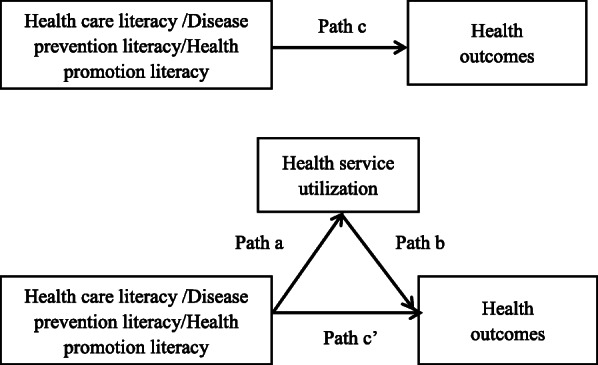
Mediator model of health literacy, health outcomes, and health service utilization

SPSS 22.0 was used for frequency analysis, reliability testing, and Pearson’s correlation analysis. Amos 22.0 was used to establish a confirmatory factor model to provide validity, construct a standardized path test, and examine the hypothesis testing results; bootstrapping was used to test the mediating effects. In the reliability analysis, the baseline value for judging the questionnaire was determined to be 0.7, with a value greater than 0.7 indicating that the questionnaire was feasible. Similarly, the Amos test of questionnaire validity was carried out using the model fit index. The specific criteria for assessment were cmin/df < 5 and GFI, AGFI, NFI, TLI, and CFI < 0.8, showing that the questionnaire had good validity. If a model has three questions, the constructed model is saturated and df is 0, and the model fit results will not be evaluated. In subsequent hypothesis testing, the collinearity test results were first used to ensure the inflation factor of the variable did not exceed 10 to show that collinearity was absent between variables. At the same time, the common-method variance test was carried out. Harman’s single-factor test was used to determine that the explanatory power of the first component in the initial eigenvalue was 42.972%, which is lower than 50%, showing that common-method variance was absent between variables. After that, path modeling and mediator model testing were conducted. The basis of the path modeling was the theoretical model (Fig. 1). Figure 1 shows that healthcare, disease prevention, and health promotion were the independent variables; health service utilization was a mediator; and health gain was the dependent variable; these were used to construct the path model.

**Fig. 2:**
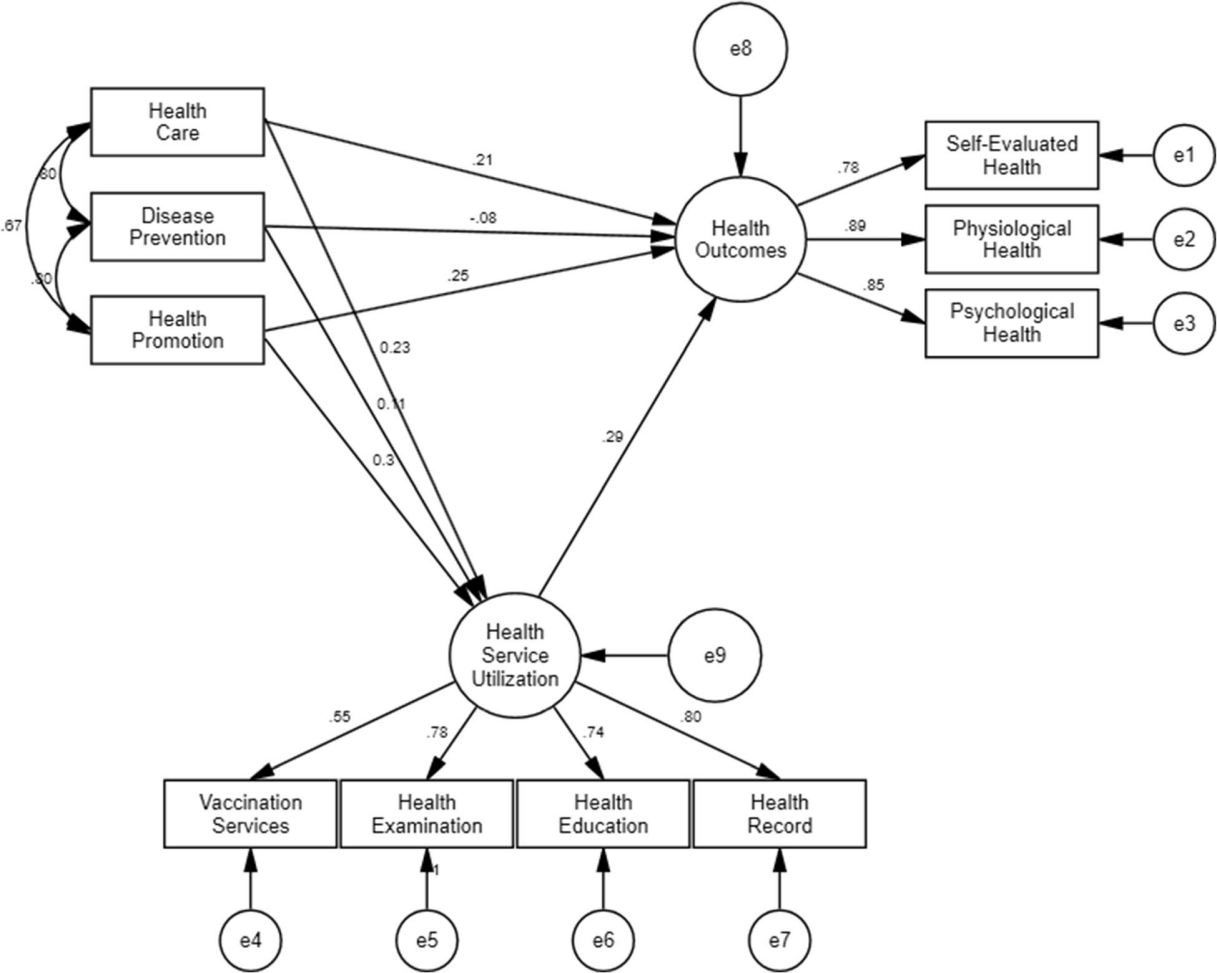
Path test of health literacy, health outcomes, and health service utilization

At the same time, bootstrapping was used for the mediator test to obtain the total, direct, and mediating effects of various variables. Mediating effect analysis needs to meet the following conditions: 1) Health literacy is significantly correlated with health outcomes (total effect; Path c, Fig. 1); 2) Health literacy is significantly related to the use of health services; 3) Control of health literacy, where health service utilization is significantly correlated with health outcomes (path b); and 4) The relationship between health literacy and health outcomes is reduced when controlling for health service utilization (indirect impact, a x b) (direct impact, pathway c’). A total of 2000 random sampling calculations were used to obtain the 95% confidence interval of the estimated value.

## Results

Table 1 shows the sociodemographic characteristics of the study participants. Among the 657 subjects, there were 244 males (37.1%) and 413 females (62.9%). The proportion of subjects aged < 40 years was 75.6, and 78.2% had an educational level of high school or below. The proportion of subjects with a bachelor’s degree was 9.4%.

**Table 1:** Demographic descriptive statistics (*N* = 657)

		Frequency	Percentage	Effective percentage	Cumulative percentage
Sex	Male	244	37.1	37.1	37.1
	Female	413	62.9	62.9	100.0
Age	30 years and below	223	33.9	33.9	33.9
	31–39 years	274	41.7	41.7	75.6
	40–49 years	135	20.5	20.5	96.2
	> 50 years	25	3.8	3.8	100.0
Education level	Primary school and below	39	5.9	5.9	5.9
	Middle school	317	48.2	48.2	54.2
	Highschool/technical Secondary school	158	24.0	24.0	78.2
	Junior college	81	12.3	12.3	90.6
	Bachelor’s degree	62	9.4	9.4	100.0

Table 2 shows the reliability analysis of the scale and the variable means. Cronbach’s α consistency coefficient was used to test internal consistency reliability, and the threshold value was 0.7. A Cronbach’s α greater than 0.7 indicated consistent reliability for that particular measurement; overall, the questionnaire was reliable. As indicated in Table 2, the analysis of health literacy showed that health promotion literacy of the floating population was better than healthcare literacy and disease-prevention literacy. Furthermore, analysis of health service utilization showed that utilization of health examination services in particular was better than utilization other types of health services. Finally, the mean value for health outcomes demonstrated that overall health of the floating population was poor, but psychological health appears better than the physiological health.

**Table 2:** Reliability coefficient, mean, and standard deviation

	Reliability coefficient		Minimum	Maximum	Mean	Standard deviation
Health literacy	.939	Healthcare	1	4	2.822	0.642
	.916	Disease prevention	1	4	2.952	0.590
	.939	Health promotion	1	4	3.035	0.608
Health services	.760	Vaccination services	1	5	2.504	1.182
		Health examination	1	5	2.763	1.374
		Health education	1	5	2.616	1.295
		Health record	1	5	2.501	1.306
Health outcome	.874	Self-evaluated health	1	5	2.282	1.050
		Physiological health	1	5	2.379	1.173
		Psychological health	1	5	2.414	1.150

Table 3 shows the validity analysis results and specific discrimination criteria. We used Amos evaluation markers CMIN/DF, NFI, IFI, TLI, CFI, and RMSEA for structural equation modeling (SEM) and model discrimination. Fit indices for health literacy, health service utilization, and health outcomes were within the acceptable range as measured by the confirmatory factor analysis of the questionnaires.

**Table 3:** Model confirmatory factor analysis

Questionnaire	CMIN/DF	NFI	IFI	TLI	CFI	RMSEA
Standard level	< 5	> 0.8	> 0.8	> 0.8	> 0.8	< 0.08
Good level	< 3	> 0.9	> 0.9	> 0.9	> 0.9	< 0.05
Health literacy	2.99	0.844	0.89	0.885	0.89	0.055
Health service utilization	2.577	0.992	0.995	0.986	0.995	0.049
Health gain	–	–	–	–	–	–

Table 4 shows the correlations between variables. Total health-service utilization was significantly positively correlated with health literacy, health outcomes, and other various dimensions. Health literacy was also significantly positively correlated with total health gain and various variables.

**Table 4:** Variable correlation

	1	2	3	4	5	6	7	8	9	10	11	12
Healthcare	1											
Disease prevention	.799^**^	1										
Health promotion	.671^**^	.800^**^	1									
Vaccination services	.340^**^	.369^**^	.353^**^	1								
Health examination	.429^**^	.426^**^	.411^**^	.413^**^	1							
Health education	.391^**^	.421^**^	.426^**^	.411^**^	.544^**^	1						
Health record	.363^**^	.368^**^	.402^**^	.419^**^	.643^**^	.605^**^	1					
Self-evaluated health	.381^**^	.359^**^	.396^**^	.234^**^	.278^**^	.275^**^	.247^**^	1				
Physiological health	.412^**^	.400^**^	.436^**^	.293^**^	.356^**^	.354^**^	.331^**^	.697^**^	1			
Psychological health	.386^**^	.355^**^	.388^**^	.233^**^	.317^**^	.348^**^	.308^**^	.669^**^	.757^**^	1		
Health service utilization	.186^**^	.182^**^	.175^**^	.490^**^	.604^**^	.581^**^	.632^**^	.165^**^	.280^**^	.259^**^	1	
Health outcome	.260^**^	.242^**^	.219^**^	.205^**^	.295^**^	.269^**^	.263^**^	.606^**^	.691^**^	.677^**^	.327^**^	1
Health literacy	.904^**^	.944^**^	.898^**^	.387^**^	.462^**^	.451^**^	.412^**^	.414^**^	.455^**^	.412^**^	.198^**^	.263^**^

**p < 0.01 (2-tailed)

Tables 5 and Fig. 2 shows the path analysis. As mentioned above, significant correlational relationships exist between various hypothesis variables; we therefore constructed a path analysis model using Amos. Table 5 shows that all paths conformed to our hypothesis *except* the paths from disease prevention literacy to health service utilization and health outcomes. Healthcare had significant positive effects on health service utilization, *r* (DF) = 0.227*,p* < 0.001. Disease prevention *did not* significantly affect health service utilization, *r* (DF) = 0.112, *p* = 0.135, nor did it significantly predict health outcomes, *r* (DF) = − 0.084, *p* = 0.25. Health promotion had a significant positive effect on health service utilization, *r* (DF) = 0.299, *p* < 0.001, and health outcomes, *r* (DF) = 0.248, *p* < 0.001. Health service utilization also had a significant positive effect on health outcomes, *r* (DF) = 0.29, *p* < 0.001, as did healthcare, *r* (DF) = 0.215, *p* < 0.001.

**Table 5:** Path test

Path	Estimate	S.Estimate	S.E.	C.R.	*P*
Health service utilization<−--healthcare	0.219	0.227	0.06	3.643	***
Health service utilization<−--disease prevention	0.118	0.112	0.079	1.493	0.135
Health service utilization<−--health promotion	0.305	0.299	0.065	4.704	***
Health outcomes<−--health service utilization	0.459	0.29	0.085	5.428	***
Health outcomes<−--healthcare	0.328	0.215	0.092	3.566	***
Health outcomes<−--disease prevention	−0.14	−0.084	0.121	−1.15	0.25
Health outcomes<−--health promotion	0.401	0.248	0.099	4.06	***

Table 6 shows the test results of confirmatory factor analysis. According to the comparison of results, the overall model of fit is appropriate, where CMIN/DF = 1.723 < 3; NFL, IFI, TLI, and CFI were all greater than 0.9, and RMSEA = 0.033 < 0.05. Therefore, the data and model match very well, and the model is valid.

**Table 6:** Model confirmatory factor analysis

Questionnaire	CMIN/DF	NFI	IFI	TLI	CFI	RMSEA
Good level	< 3	> 0.9	> 0.9	> 0.9	> 0.9	< 0.05
Resulting value	1.723	0.987	0.995	0.991	0.995	0.033

Table 7 shows the mediation test. We tested the significance of this indirect effect using bootstrapping procedures. Unstandardized indirect effects were computed for each of the 2000 bootstrapped samples, and the 95% confidence interval was computed by determining the indirect effects at the 2.5th and 97.5th percentiles. The mediation test was significant for healthcare effects on health outcomes through health service utilization, as the bootstrapped unstandardized indirect effect was 0.280, and the 95% confidence interval ranged from 0.174–0.378. Thus, the indirect effect was statistically significant. Furthermore, the direct effect was 0.215, and the 95% confidence interval ranged from 0.108–0.313, indicating a true direct effect. Finally, the mediating effect size was 0.066, with a 95% confidence interval of 0.023–0.299, indicating a partial mediation model. Hence, the results show that the total effect, direct effect, and mediating effect of disease prevention on health outcomes through health service utilization were *not* true, indicating that health service utilization was not a mediator variable for the effects of disease prevention literacy on health outcomes. However, the mediation test was significant for health promotion on health outcomes through health service utilization, with a total effect size of 0.335, and a confidence interval ranging from 0.192–0.467, indicating that the total effect is true. The direct effect size was 0.248, with a confidence interval of 0.101–0.389, indicating a true direct effect. Finally, the mediating effect size was 0.087, with a confidence interval of 0.047–0.133, indicating that the total effect is true, and the model is a partial mediation model.

**Table 7:** Mediation effects test

Path	Effects	Effect size	S.E.	*P*-value	LLCI	ULCI
Healthcare→health service utilization→health outcomes	Total effects	0.28	0.051	0.001	0.174	0.378
	Direct effect	0.215	0.052	0.001	0.108	0.313
	Mediating effects	0.066	0.022	0.001	0.023	0.299
Disease prevention→health service utilization→health outcomes	Total effects	−0.051	0.08	0.112	− 0.206	0.112
	Direct effect	−0.084	0.08	0.32	−0.236	0.078
	Mediating effects	0.033	0.021	0.112	−0.007	0.075
Health promotion→health service utilization→health outcomes	Total effects	0.335	0.07	0.001	0.192	0.467
	Direct effect	0.248	0.072	0.001	0.101	0.389
	Mediating effects	0.087	0.022	0.001	0.047	0.133

## Discussion

This study employed SEM to examine the relationship between health literacy and health outcomes in China’s floating population and simultaneously analyzed the mediating effects of health service utilization. Health literacy affected health outcomes through two dimensions—healthcare literacy and health promotion literacy—but disease prevention did not directly affect health outcomes. Notably, health literacy had positive effects on health outcomes, and health service utilization had partial mediating effects between health literacy and health outcomes.

Healthcare literacy illustrates whether people are equipped with an understanding of and ability to communicate about health services and whether they possess medical skills for emergencies; healthcare literacy directly affects health outcomes. Notably, the majority of individuals in the floating population choose to either self-medicate or forgo medical treatment. One out of 5 individuals reported the choice to visit private/individual clinics or community health service stations/rural health centers. Finally, a small minority choose to seek care at a county-level hospital [29]. Such choices are the result of interactions between subjective and objective factors and are associated with education level, income, occupation, health status, and disease. Self-medication is typically the first choice among individuals in the floating population after disease onset; this requires the individual to actively search for treatment information. If they are unable to self-medicate, they could suffer from a major disease and must seek professional medical services. This requires them to be able to communicate with physicians and understand medication instructions, among other aspects of treatment. Furthermore, income and social security limit the ability of the floating population to access medical services. They must therefore master certain self-treatment techniques, such as traditional massage, acupuncture, and cupping therapy.

Health promotion literacy directly affects health outcomes. Health promotion refers to a social behavior or strategy that uses administrative or organizational measures to coordinate various social departments, communities, families, and individuals to carry out individuals’ health responsibilities to jointly maintain and promote health. In health literacy, health promotion refers to an individual’s understanding of the factors affecting physical and mental health, such as governmental health policies, community facilities, social networks, work environments, and residential environments. It also refers to actively searching for relevant health education resources and making decisions about improving health. To better satisfy the health service needs of the floating population and improve their health levels, the government can rely on multicenter governance theory and social policy development directions to ask communities to construct a more comprehensive health service utilization system. Such a system would include three major providers of floating population health services: community residential (village) committees, community health service centers, and social workers/community organizations [30]. The living conditions and work environments of the floating population are generally poor, and these adverse factors have negative effects on health. Notably, the effects of adverse work environments are particularly prominent. Although participation in social activities, staying with family members, and socializing with local employees have important positive effects on promoting health among the floating population. Therefore, relevant departments should employ the necessary measures to improve the living conditions and work environments of the new generation of rural workers to improve their health [31].

Disease prevention did not directly affect health outcomes. Disease-prevention health literacy mainly refers to understanding the importance of health behaviors and health examinations, and it entails understanding the effects of smoking, low exercise intensity, and excessive drinking; knowing that vaccinations and health examinations can help prevent disease; and possessing the ability to improve health behaviors [32, 33]. Workers should also be able to decide how to protect themselves from disease based on suggestions from friends, family, and media. In this regard, this study’s results conflict with previous findings [34]. It could be that the floating population is one that is naturally selected for health, and, generally, only healthy people can migrate. Most individuals in the floating population believe that “health” means the absence of disease; hence, the absence of obvious symptoms indicates a healthy body, so they may pay little attention to disease prevention information [35]. Meanwhile, most of China’s floating population were originally farmers and manual laborers; their low educational levels may limit their understanding of nutrition, healthcare, and disease prevention. Furthermore, under profit maximization and industry competition pressure, small and medium-sized manual-labor enterprises may choose to increase manufacturing speed to produce more products. They employ a piece-rate payment system to encourage workers to work longer hours. This not only affects their health but also reduces normal rest and leisure time, which includes time for exercise and attention to diet. Long working hours, poor living conditions, poor work environments, and stress related to integration cause members of the floating population to lack the energy needed to gain disease prevention knowledge and cultivate healthy lifestyle habits [36]. Most of the floating population is in the most active period of economic activity of their life (in the age group of 18–49 years), and they will spend two-thirds of their time working. Therefore, in addition to the government’s promotion of the importance of disease prevention literacy to health, the floating population should also understand the concept of prevention over treatment through the construction of healthy enterprises [37].

Health service utilization directly affects health outcomes. In 2016, China proposed the “Healthy China” strategy, establishing public health as a primary objective. However, data from the “2016 China Floating Population Development Report” showed that the health of the floating population, which is an important component of the labor force, is declining [38], highlighting the need to provide medical, preventive, healthcare, and rehabilitation services for this population. Studies have shown that different household registrations affect the preventive health services provided by public health departments. To enable the floating population to obtain basic public health services, it is necessary to eliminate the separation between rural- and urban-registered households, increase the permanent residence rate of the floating population, promote a basic health security system for the entire country, and strengthen employment training and education levels [39]. This requires synergistic effects from top and middle governments as well as grassroots involvement. After the top government integrates health into public policies, middle governments need to expand the structural intervention of public health services for health promotion. This also requires the government to focus on providing public health services and strengthening the municipal level as a health-related decision structure [40]. The grassroots level should refer to community pharmacies in the US, which can intervene to help prevent primary diseases through smoking cessation, weight management programs, needle exchange, and vaccination services, among others [41]. Moreover, the floating population does not passively receive public services, and their participation (PPI) has become an indispensable part of healthcare, with a focus on improving their participation in public affairs [42]. Research has also found that the effects of health service announcements are better received after people view health-related fictional programs [43]. Thus, public health media should not only utilize new media channels but also find ways to attract audiences and help them gain health knowledge. Lastly, health impact evaluation is a typical task in public health services. A study in Germany found that health assessment can clarify the responsibilities of health services [44]. Therefore, assessing the impact of various types of health services on health for resource matching is an additional option.

Health service utilization had partial mediating effects in the relationship between healthcare literacy and health promotion literacy and health outcomes. Health service utilization was not found to have a mediating effect in the relationship between disease prevention literacy and health outcomes. The effects of health literacy on health outcomes can be partially explained by health service utilization. For example, females with lower health literacy tend to use less preventive healthcare services, including flu vaccination and cervical and breast cancer screening [45]. Moreover, low health literacy is more common among elderly people [46]. A lack of health literacy also directly affects the effective utilization of health services and social welfare by chronic disease patients and is closely associated with disease management and health outcomes [47]. Therefore, good health literacy in the floating population can enable them to clearly understand, recognize, and control the relationship between their lifestyle and health status [48]. Improving health knowledge, changing unhealthy lifestyle and work habits (e.g., working overtime or working when sick), actively using various available health resources, and making a habit of regular physical exams can form a positive feedback loop of early diagnosis, treatment, and recovery. In summary, the floating population poses a top health priority, and the establishment of a national health management system should focus on family health advocacy. Regarding the fact that health service utilization does not have mediating effects in the relationship between disease prevention and health outcomes, the root cause is that disease prevention literacy did not affect health outcomes. This is because the floating population comprises mostly young people who have poor health-risk awareness. Meanwhile, disease prevention literacy involves professional preventive medical knowledge. Due to educational limitations, it is difficult for the floating population to have such understanding. In the future, subjects can be stratified by education level to examine the effects of disease prevention literacy on health outcomes. Given the current COVID-19 pandemic, the mobility of the floating population poses a huge health risk. Hence, there is an urgent need to strengthen their health literacy regarding disease prevention to reduce the risk posed by COVID-19.

This study has some limitations. First, the health literacy scale used in this study is a typical scale for critical health literacy, but there is a lack of scales for functional health literacy and communicative health literacy. In the future, the All Aspects of Health Literacy Scale (AAHLS) can be used to measure health literacy in the floating population as it is suitable for evaluating functional, communicative, and critical health literacy. In addition, there is a risk of bias when a self-evaluated health literacy tool is used since widespread optimism/pessimism and memory deficiencies can affect the outcomes. Second, the cross-sectional design did not allow for causality deduction. Conducting longitudinal studies and reassessing health outcomes will help identify causality in health literacy. Moreover, the samples came from three prefecture-level cities. The educational levels of floating populations in different parts of China are different, which affects health literacy. This could have affected sample representativeness and the generalization of the results. Lastly, we did not investigate the connection between health literacy and health behavior. Examining this relationship in the future could aid in designing intervention measures at the individual level, thereby improving the health of vulnerable populations.

## Conclusion

This study found that health service utilization had partial mediating effects between health literacy and health outcomes. Health literacy affects the proactiveness of the floating population in health service utilization through healthcare literacy and health promotion literacy, thereby affecting health outcomes. Improving health literacy in the floating population will help improve their health outcomes. Health service providers need to enhance the diversity of health services and ensure that the floating population has the necessary external conditions to improve their individual health.

## Data Availability

The datasets used in this study can be obtained from the corresponding author upon reasonable. request.

## Abbreviations

HLS: Health literacy survey
PPS: Probability proportional to size
SF: Short form
PHQ: Patient health questionnaire
HSCL: Hopkins symptoms check list
SEM: Structural equation modeling
PPI: Participation
AAHLS: All aspects of health literacy scale
CMIN: Chi-square
NFI: Normed fit index
IFI: Incremental fit index
TLI: Tucker-lewis index
CFI: Comparative fit index
RMSEA: Root-mean-square error of approximation
LLCI: Lower level of confidence interval
ULCI: Upper level of confidence interval
